# L-Arginine-eNOS-NO Functional System in Brain Damage and Cognitive Impairments in Cerebral Small Vessel Disease

**DOI:** 10.3390/ijms241914537

**Published:** 2023-09-26

**Authors:** Larisa A. Dobrynina, Alla A. Shabalina, Kamila V. Shamtieva, Elena I. Kremneva, Maryam R. Zabitova, Marina V. Krotenkova, Anastasiia G. Burmak, Elena V. Gnedovskaya

**Affiliations:** Research Center of Neurology, 80 Volokolamskoe Shosse, 125367 Moscow, Russia; dobrla@mail.ru (L.A.D.); ashabalina@yandex.ru (A.A.S.); kamila.shamt@gmail.com (K.V.S.); moomin10j@mail.ru (E.I.K.); krotenkova_mrt@mail.ru (M.V.K.); burmak_n@mail.ru (A.G.B.); gnedovskaya@mail.ru (E.V.G.)

**Keywords:** cerebral small vessel disease, cognitive impairments, NO bioavailability, NO deficiency, L-arginine, erythrocyte disaggregation rate, BBB permeability, dynamic contrast-enhanced MRI

## Abstract

Cerebral small vessel disease (CSVD) is a significant cause of cognitive impairment (CI), disability, and mortality. The insufficient effectiveness of antihypertensive therapy in curbing the disease justifies the search for potential targets for modifying therapy and indicators supporting its use. Using a laser-assisted optical rotational cell analyzer (LORRCA, Mechatronics, The Netherlands), the rheological properties and deformability of erythrocytes before and after incubation with 10 μmol/L of L-arginine, the nitric oxide (NO) donor, blood–brain barrier (BBB) permeability assessed by dynamic contrast-enhanced MRI, clinical, and MRI signs were studied in 73 patients with CSVD (48 women, mean age 60.1 ± 6.5 years). The control group consisted of 19 volunteers (14 women (73.7%), mean age 56.9 ± 6.4 years). The erythrocyte disaggregation rate (y-dis) after incubation with L-arginine showed better performance than other rheological characteristics in differentiating patients with reduced NO bioavailability/NO deficiency by its threshold values. Patients with y-dis > 113 s^−1^ had more severe CI, arterial hypertension, white matter lesions, and increased BBB permeability in grey matter and normal-appearing white matter (NAWM). A test to assess changes in the erythrocyte disaggregation rate after incubation with L-arginine can be used to identify patients with impaired NO bioavailability. L-arginine may be part of a therapeutic strategy for CSVD with CI.

## 1. Introduction

Cerebral small vessel disease (CSVD), associated with age and vascular risk factors, is the main cause of vascular cognitive impairment, mixed variants of Alzheimer’s disease, and is a significant cause of stroke, disability, and mortality [1,2,3,4,5].

Arterial hypertension (AH) is the main risk factor for age-related CSVD [5,6,7]. However, a strategy to prevent dementia through standard and aggressive control of hypertension has shown inconsistent results [8,9,10]. The lack of CSVD-modifying treatments justifies the search for “druggable” targets, based on pathogenic pathways [5]. Since endothelial dysfunction, characterized by reduced NO bioavailability/NO deficiency, is a prerequisite for CSVD, finding ways to evaluate the main functional system of NO synthesis—L-arginine-eNOS-NO—is justified [11,12,13]. Maintaining the balance of this functional system may be a potential target of CSVD therapy.

Under normal conditions, nitric oxide synthase (NOS) catalyzes the conversion of arginine, O_2_, and electron transfer of nicotinamide adenine dinucleotide phosphate (NADPH) to form NO and citrulline in the presence of tetrahydrobiopterin (BH4) and other cofactors [12]. Reduced NO bioavailability/NO deficiency is most commonly cited in association with decreased endothelial NOS (eNOS, NOS3) activity in endothelial dysfunction. Reduced NO bioavailability/NO deficiency leads to a vasospastic, prothrombotic inflammatory status of the vascular wall, associated with the main CSVD mechanisms—hypoxia/ischemia and increased blood–brain barrier (BBB) permeability [11,12,14].

Impairments in other parts of the functional system of NO synthesis—a relative deficiency of arginine or BH_4_—lead to a decrease in NO synthesis through an increase in unbound NOS and its uncoupling, and a shift in oxidation to molecular oxygen with the formation of O_2-_ [15,16]. Among the causes of relative arginine deficiency are competitive relationships with arginase for NOS and inhibition of NOS by arginine analogues, primarily methylarginines [17,18]. Increased levels of the latter inhibit L-arginine transport into the endothelial cell, which leads to the arginine paradox—dependence of cellular NO production on exogenous L-arginine when NOS is saturated with intracellular L-arginine [17,19].

The dependence of NO production on exogenous L-arginine became the basis for research on the use of the latter as a drug that improves endothelial function in a number of diseases and aging [20,21,22,23]. Despite the promising prospects of the results of most studies, arginine has not found wide application in official medicine. An obvious need is to determine the optimal target group, based on indicators justifying L-arginine therapy to ensure NO bioavailability and prevent NOS uncoupling [18]. The hypothesis of the feasibility of long-term oral administration of high doses of L-arginine to slow down CSVD progression and its manifestations was proposed more than 10 years ago [24]. However, it has not been developed to date.

The rheological properties and deformability of erythrocytes can potentially be used for assessment of the L-arginine-eNOS-NO functional system and identification of CSVD patients with reduced NO bioavailability/NO deficiency who are potential candidates for L-arginine therapy. The choice of erythrocytes as an individual assessment model was justified by the presence of eNOS, involved in the synthesis, transport, and release of NO and adenosine triphosphate (ATP) metabolic products, the active regulation of erythrocyte deformability and blood fluidity, and the control of systemic NO bioavailability, endothelial state, and vascular tone [25,26,27,28].

The contribution of altered erythrocytes’ rheological properties and deformability has been described in many cardiovascular diseases, such as AH [29], coronary heart disease [30], and diabetes mellitus (DM) type 2 [31]. Among the pathological mechanisms of diseases and their complications mediated by erythrocytes with altered functional properties, there is a deterioration of perfusion, endothelial cell dysfunction, and others [30,31,32]. The high significance of the altered functional properties of erythrocytes in the development of CSVD is confirmed in experimental animals. In spontaneously hypertensive stroke-prone rats, recognized as the most relevant model of CSVD, the first stage of pathology development is erythrocyte stasis, which subsequently becomes widespread and leads to ischemia and high BBB permeability, the main mechanisms for CSVD development [33].

In the present study, the obtained erythrocyte rheological properties and deformability characteristics before and after incubation with L-arginine, an NO donor, were used to obtain individual indicators of NO bioavailability. The predictive value of these indicators was clarified by their relationship with the severity of cognitive impairment (CI) and diagnostic MRI signs of CSVD.

It has been established that reduced NO bioavailability/NO deficiency is one of the conditions for an increased BBB permeability, as the leading mechanism for the development of CSVD [34]. In this regard, the associations of the erythrocytes’ aggregation and deformability changes before and after incubation with L-arginine with BBB permeability, according to T1-dynamic contrast-enhanced MRI (DCE-MRI) data and the level of circulating markers (tumor necrosis factor alpha (TNF-α), transforming growth factor beta-1 (TGF-β1), vascular endothelial growth factor-A (VEGF-A), hypoxia-inducible factor 1-alpha (HIF-1α), fibroblast growth factors (FGFs), plasminogen activator inhibitor (PAI-1), and tissue plasminogen activator (t-PA)), were further clarified.

DCE-MRI is largely used in the study of CSVD pathophysiology [35]. The main indicators of BBB permeability are the contrast agent transfer coefficient from blood plasma to the brain (Ktrans), the fractional blood plasma volume (Vp, corresponding to the blood plasma volume per unit of brain volume), and the area under the contrast curve (AUC, corresponding to contrast agent retention in the brain) [36,37]. Recently, the relation of increased BBB permeability with damage to normal-appearing white matter (NAWM) and the subsequent development of white matter hyperintensity (WMH) [38,39], the severity of WMH [39,40], and the development of vascular dementia [41] were established in patients with CSVD.

### Objective

To assess the state of the L-arginine-eNOS-NO functional system in CSVD patients based on the properties of erythrocytes after incubation with L-arginine and the role of deviance in brain damage and CI.

## 2. Results

The rheological properties of erythrocytes before and after incubation with L-arginine were assessed (Table 1).

The rheological properties of erythrocytes before and after incubation with L-arginine for most parameters showed significant differences between the CSVD patients and controls, with unidirectional changes in the studied parameters. The significance of the resultant (after incubation with L-arginine) rheological characteristics was assessed by the AUC, the threshold with optimal sensitivity and specificity, and the odds ratio (Table 2).

Youden’s test showed that y-dis had the threshold value with the highest sum of sensitivity and specificity, compared to the other parameters. Its threshold value was used to divide the subjects into two groups, with y-dis < 112 s^−1^ and >113 s^−1^.

The resulting groups differed in terms of vascular risk factors, severity of cognitive deficits, and the WMH, which is the main MRI sign of CSVD (Table 3).

Patients with severe AH, DM type 2, obesity, WMH Fazekas scores of 2–3, and greater CI predominated in the y-dis group above the threshold value (>113 s^−1^). Clinical characteristics of CSVD patients and controls are presented in the Appendix (Table A1).

The groups were compared in terms of the BBB permeability values obtained by DCE-MRI (Table 4).

The BBB permeability by AUC in the y-dis > 113 s^−1^ group compared to the y-dis < 112 s^−1^ group was higher in GM and NAWM. Data on the BBB permeability by DCE-MRI in CSVD patients and controls are presented in the Appendix (Table A2).

The groups were compared for the plasma levels of vascular damage markers, nitric oxide, and its metabolites (Table 5). The matched groups differed only in the higher t-PA level in the group with y-dis > 113 s^−1^.

A comparison of the indicators of vascular damage, nitric oxide, and its metabolites in CSVD patients and controls is presented in the Appendix (Table A3).

## 3. Discussion

The study was devoted to the investigation of the role of the impaired L-arginine-eNOS-NO functional system in CSVD based on the search for individual indicators of its state. The validity of the study is determined by the priority of endothelial dysfunction in the initiation and progression of CSVD associated with reduced NO bioavailability [11,12,13]. The presence of indicators signaling individual disorders in the L-arginine-eNOS-NO system with reduced NO bioavailability/NO deficiency is extremely valuable, considering the lack of treatment modifying the course of CSVD [5] and the possibility of using the NO donor, L-arginine, as a therapeutic agent [42,43,44], including in elderly patients with AH and CI [23]. Despite the proven dependence of NO synthesis on exogenous L-arginine [17,19] and its effectiveness in small patient samples in pathologies with endothelial dysfunction [23,42,43,44], L-arginine intake cannot be a priori uncontrolled. This is due to its complex metabolism and unpredictable consequences when used in clinical practice [24], such as the potential to switch from an eNOS to an iNOS pathway [45] and lead to NO excitotoxicity. In addition, the lack of effect of L-arginine intake in some studies also indicates the need to develop indications for its use and search for individual sensitive indicators of reduced NO bioavailability.

The choice of erythrocytes as a model for assessing individual NO bioavailability disorders was predetermined by the presence of eNOS, involved in the synthesis, transport, and release of NO and ATP metabolic products, regulation of erythrocyte deformability and blood fluidity, control of systemic NO bioavailability, and vascular tone [25,26,27,28]. It is likely that the L-arginine-eNOS-NO functional system of erythrocytes is even more vulnerable and unstable than the endothelium. This is because erythrocytes are the earliest and most accessible target for reactive oxygen species. The varied participation of erythrocytes in the redox cycle during oxygenation and deoxygenation of hemoglobin supports their oxidative damage and inflammatory responses [46]. This is associated with the maintenance of eNOS dissociation and NO deficiency, and the decreased membrane fluidity with increased stiffness, which underlie microcirculatory disorders [46].

The selection of erythrocyte deformability, aggregation, and disaggregation properties to identify persons with NO deficiency was justified by the high significance of these changes in erythrocyte properties in the development of CSVD. In an experiment on a relevant CSVD model of spontaneous hypertensive rats prone to stroke, it was found that erythrocyte accumulations in capillaries and arterioles are the first manifestation of the CSVD, the severity of which increases with the aging of animals [33,47]. It is this pathological event that initiates vascular wall damage and the subsequent cascade of events that make up the morphological basis of CSVD—high BBB permeability, microbleeds and micro-thrombosis, and cerebral infarctions due to arteriole occlusion [33,47].

We used the resulting (after incubation with L-arginine) rheological characteristics and erythrocyte deformability to calculate the area under the curve, odds ratio, and optimal sensitivity and specificity. The elongation of the erythrocyte disaggregation rate (y-dis) was the best among the evaluated parameters in determining the threshold values for CSVD. This is also consistent with the above-described phenomenon of erythrocyte accumulation in capillaries and arterioles, as initiating and supporting CSVD progression [33]. The division of participants according to the threshold level made it possible to establish a relationship between the increased erythrocyte disaggregation time (y-dis) and the severity of AH, DM type 2, and obesity.

Although previous studies have shown that all these risk factors can independently influence the rheological properties of erythrocytes [29,48,49], the relationships we have established also indicate the particular importance of reduced NO bioavailability/NO deficiency for CSVD development. There is a lack of a strict cause-and-effect relationship between the severity of vascular risk factors and the severity of CSVD. This is the main explanation for the lack of the expected reduction in the prevalence of CI due to CSVD with appropriate control of AH and other vascular risk factors [8,9,10]. Our data indicated that reduced NO bioavailability/NO deficiency in CSVD patients could be a special condition for the realization of age-dependent vascular risk factors’ pathological potential. This assumption is also supported by experimental data in eNOS-deficient mice. Initially, eNOS-deficient mice were used as a model for studying spontaneous AH [50,51], and later as a relevant CSVD model [52]. NO knockout mice reproduce all vascular risk factors and mechanisms for CSVD development—from AH and systemic aspects of the metabolic syndrome, such as insulin resistance and hyperlipidemia [53], to widespread thrombotic microangiopathy with the formation of infarcts and high BBB permeability [52]. Thus, the study allowed us, based on changes in the properties of erythrocytes that have eNOS similar to that of the endothelium, to obtain evidence of the connection between NO deficiency and vascular risk factors, clinical manifestations, and mechanisms of CSVD development.

Patients who exceeded the erythrocyte y-dis threshold after incubation with L-arginine had significantly lower cognitive scores on the MoCA scale, and higher WMH and BBB permeability in NAWM and GM, assessed by AUC corresponding to the retention of the contrast agent in the brain parenchyma. This made it possible to consider exceeding the threshold values of erythrocyte y-dis after incubation with L-arginine as equivalent to a functional NO deficiency, and points to erythrocytes as a significant participant in pathogenetic processes in CSVD. The established relationship for this indicator can be explained from the perspective of capillary dysfunction [54,55], and accompanying neurovascular unit dysfunction. These processes are the cause of deterioration of perfusion, inefficient metabolism, and disruption of BBB integrity [55,56], and they explain the observed relationships between a slow erythrocyte disaggregation rate after incubation with L-arginine and increased BBB permeability, WMH, and CI severity.

Additionally, the levels of circulating markers associated with vascular wall damage were compared between groups with different NO bioavailability. We believed that their profiles may indicate additional conditions supporting the pathological mechanisms in reduced NO bioavailability/NO deficiency disorders. Among all the parameters studied, only the increase in t-PA in the y-dis > 113 s^−1^ group was significantly different from the y-dis < 112 s^−1^ group. T-PA, along with its well-known role in fibrinolysis, has pleiotropic activities in the central nervous system—neuroplasticity, excitotoxicity, and influence on BBB permeability [57]. Previously, we established a relationship between increased t-PA levels and MRI changes associated with high BBB permeability [58]. However, there are reasons to believe that the combined effect of t-PA, NO, and its oxidation products provides a continuum of vascular and cerebral damage associated with neurodegeneration and mixed vascular-degenerative forms of the CSVD. Therefore, in an experiment on t-PA-deficient mice, it was found that BBB damage alone is not sufficient to cause neurodegeneration—a subsequent ONOO-mediated event is required. Results indicated that BBB damage associated with neurodegeneration occurs if NO and ONOO excitotoxicity are mediated by t-PA [59].

## 4. Materials and Methods

### 4.1. Study Participants

This study included patients aged 46–70 years with cognitive and other cerebral complaints, as well as brain MRI changes corresponding to CSVD (WMH, lacunes, enlarged perivascular spaces, microbleeds, and cerebral atrophy) [60]. Patients with a low WMH burden (Fazekas scale score of 1) were included in the study if they had AH stage 2 or 3 and/or ≥1 lacuna.

Exclusion criteria: (1) CI due to probable Alzheimer’s disease according to the U.S. National Institute on Aging criteria [61,62]; (2) patients with small subcortical infarcts/lacunes <3 months after stroke; (3) CSVD due to other independent causes (genetic, inflammatory, thrombophilic, systemic, toxic, history of severe migraines); (4) a different cause of stroke and concomitant brain pathology other than CSVD; (5) >50% atherosclerotic stenosis of the extra- or intra-cranial arteries; (6) serious medical condition—cardiac (ejection fraction < 50%), endocrine (diabetes mellitus (DM) type 1 or 2 with severe vascular complications, uncompensated thyroid disorder), renal (chronic kidney disease with glomerular filtration rate < 30 mL/min), etc., and (7) contraindications for MRI.

The control group consisted of volunteers with no clinical or MRI evidence of vascular and degenerative brain pathology.

According to the above criteria, the main study group included 73 CSVD patients (48 women (65.8%), mean age 60.1 ± 6.5 years) who met the inclusion and exclusion criteria for this study. The control group consisted of 19 volunteers (14 women (73.7%), mean age 56.9 ± 6.4 years).

The study was approved by the Local Ethics Committee of the Research Center of Neurology, Protocol No. 12-3/16, dated 14 October 2016. All subjects provided written informed consent. All the methods were carried out in accordance with the Declaration of Helsinki.

Traditional vascular risk factors, such as AH (determined by anamnestic and taking into account the daily monitoring of blood pressure, carried out by all participants) [63], hypercholesterolemia, obesity, DM, and smoking, were assessed in the patients and controls.

Cognitive impairment was assessed using the Montreal Cognitive Assessment (MoCA) scale [61].

Imaging was carried out in a Siemens MAGNETOM Verio 3T scanner (Siemens Medical Systems, Erlangen, Germany) with a standard 12-channel matrix head coil. Two neuroradiologists (E.I.K. and M.V.K.) evaluated the brain MRI studies in a standardized manner and blinded to clinical information.

The Fazekas scale [64] was used to quantify T2 FLAIR WMH (score 0–3) as well as semi-automatic WMH segmentation using the LST toolbox (http://www.applied-statistics.de/lst.htm, accessed on 20 July 2023) for SPM12 (http://www.fil.ion.ucl.ac.uk/spm, accessed on 20 July 2023), with further manual correction using the ITK-SNAP viewer (http://itksnap.org, accessed on 20 July 2023). The obtained data were saved as a binary mask, which was taken into consideration when a NAWM mask was subsequently created to calculate the BBB permeability.

### 4.2. MRI Study and Data Analysis

DCE-MRI was performed for the BBB leak assessment. After two T1 volumetric interpolated breath-hold examination (T1-VIBE) acquisitions (flip angles 2 and 15) for pre-contrast T1 maps, we injected gadodiamide (Omniscan; GE Healthcare) 0.2 mL/kg (i.e., 0.1 mmol/kg body weight) at a rate of 3 mL/s, intravenously via an injection pump, and then repeated the 3D T1-weighted sequence sequentially 100 times for 15 min and 33 s. The scanning parameters were: TR—8.6 ms, TE—4 ms, field of view—250 mm, matrix—256 × 230 pixels, flip angle—15 degrees, and slice thickness—3.6 mm.

The entire dataset underwent preliminary processing using the NordicNeuroLab software (NordicICE, Norway). This included automatic correction of motion artefacts, correction of pre- and post-contrast data in the dynamic series, and concentration of the contrast agent in the brain tissue calculation using the relative signal change and T1 mapping. Individual vascular input functions were derived semi-automatically from the superior sagittal sinus [40]. The hematocrit, contrast agent dose, and relativity of the contrast agent were set individually for each patient. The Patlak pharmacokinetic model was used to assess the BBB permeability in CSVD, resulting in Ktrans, Vp, and AUC maps.

Once the permeability parameter maps were obtained, further data processing was performed in SPM12 (http://www.fil.ion.ucl.ac.uk/spm, accessed on 20 July 2023). This included the following steps: co-registration of each subject’s permeability parameter maps and the T1 images, and segmenting the T1 images into grey matter and white matter, followed by correction of the obtained images using WMH masks based on a MATLAB script (https://matlab.ru/, accessed on 20 July 2023), resulting in the binary images of the corrected grey and white matter. The permeability parameters were separately calculated in ITK-SNAP (http://itksnap.org, accessed on 20 July 2023) for the grey matter (GM), NAWM, and WMH by superimposing the relevant masks over the individual permeability maps.

### 4.3. Laboratory Research

The rheological properties of erythrocytes were studied using a laser optical rotary cell analyzer (LORRCA, Mechatronics, The Netherlands). The LORRCA (LORRCA^®^) is a unique instrument that combines red blood cell (RBC) deformability with ektacytometry, osmoscan, and aggregometry, all temperature controlled. It is capable of fully automated measurement and calculation of various phenomena of RBCs via analysis of their rheological behavior. The technique accurately detects deformability as a function of shear stress and aggregation of the RBCs [65,66].

LORRCA is supplied with completely automated management and software. In accordance with the LORRCA instructions, samples of whole venous blood (anticoagulant EDTA K3) obtained during cubital venipuncture were used for the study, which were stored for no more than 60 min before the study at room temperature.

LORRCA incorporates a series of techniques to perform specific measurements:

- Deformability—laser diffraction ektacytometry, parameterization of the deformation curve, cell and cell membrane stability, and deformability under an osmotic gradient.
- Aggregation and disaggregation—syllectometry, with the extent of aggregation, aggregation kinetics, and tendency [67].

For the erythrocyte deformability study, 25 µL of whole venous blood with EDTA K3 was gently mixed by inverting the tube about 30 times with 5 mL of polyvinylpyrrolidone buffer (Mechatronics, Hoorn, The Netherlands). This analyzer implements the method of recording the intensity of backscattering from a blood sample placed between two coaxial glass cylinders to create a simple shear flow [68]. A thin layer of erythrocyte suspension is distributed between two concentric cylinders. The rotation of the outer cylinder causes deformation (elongation) of the erythrocytes. With the help of a video camera, the diffraction indices of the laser beam, which fixes this deformation, are taken, followed by computer analysis of the obtained data. The program for evaluating the erythrocyte elongation index displays quantitative data on the stress dependence (deformability curve) and the correlation from the time of exposure to this stress (stability curve). With the simultaneous analysis of the two tests, data were obtained on the stability of erythrocyte membranes. The software performs a fully automated analysis of the deformability of erythrocytes and provides internal control of the stability of the parameters of the measurements. The LORRCA has the capability to automatically measure RBC deformability over a gradient of osmotic values. This results in a continuous curve. For the erythrocyte aggregation and disaggregation study, 1 mL of whole venous blood was placed in a preheated (37 °C) Couette system consisting of two cylinders. A photodiode embedded in a fixed inner cylinder detects the intensity of backscattered light during the aggregation and disaggregation of erythrocytes. Initially, the rotation of the cylinders causes flow to occur in the thin layer of the sample erythrocyte suspension, which causes separation of aggregates formed under static conditions. Measurements of the spontaneous aggregation kinetics are performed after an abrupt stop in the cylinder rotation. There is a restoration of the erythrocytes’ shape after deformation in the shear flow and their spontaneous aggregation, in which two stages are distinguished—a faster stage of formation of two-dimensional aggregates, “coin columns”, and a smoother stage of formation of three-dimensional aggregates. The next step of the analysis involves a stepwise increase in the shear rate, from 0 s^−1^ to ~900 s^−1^. This procedure is accompanied by erythrocyte disaggregation and makes it possible to determine the shear rate at which complete disaggregation is achieved and no new aggregates are formed. This parameter characterizes the strength of erythrocyte aggregates. The syllectogram curve is automatically built at the end of the analysis [67,68,69].

The mathematical model describes these processes by calculating such parameters as the times of the first and second phases of aggregation (Ts and Tf), as well as the aggregation amplitude (Amp), showing the overall degree of aggregation, the aggregation index (AI), calculated as an integral of the overall syllectogram curve, and y-dis, reflecting the force required to destroy erythrocyte aggregates (Table 6).

To study the effect of NO donors on the aggregation, disaggregation, and deformability of erythrocytes, samples were incubated with L-arginine at a final concentration of 10 M at a temperature of 37 °C for 30 min, and then the rheological properties were re-evaluated. To prepare the solution, L-arginine powder was dissolved in distilled water. To study the effect of L-arginine in vitro, whole blood samples were divided into two aliquots: L-arginine was added to one, and distilled water in the same amount was added to the second—a control sample. Both aliquots were incubated under the same conditions, and then the RBC properties were studied in both samples for subsequent comparisons.

TGF-β1, FGFs, and VEGF-A were determined using BCM Diagnostics reagent kits, HIF-1α by Cusabio, TNF-α by Invitrogen, and t-PA and PAI-1 by Technoclone. Calibrators from the reagent manufacturers were used in all tests. Measurements were carried out in parallel on Perkin Elmer (USA) VICTOR2 and Real-Best (Russia) plate readers using lyophilized control sera/plasma with low and high contents of the studied parameters.

### 4.4. Statistical Analysis

Statistical analysis was performed using IBM SPSS 23.0 (IBM SPSS Statistics, version 23.0, IBM Corp., Armonk, NY, USA) and R 3.4.3 (R Foundation for Statistical Computing, Vienna, Austria) software. Data were presented as n (%) for categorical variables or as mean ± standard deviation (SD) or median [interquartile range (IQR)] for quantitative data. Differences between groups were determined using χ^2^, the independent samples t-test, univariate analysis of variance, or the Kruskal–Wallis test, where appropriate. In all cases, two-way statistical criteria were used. The null hypothesis was rejected at *p* < 0.05. ROC (receiver operator characteristic) analysis, with determination of the threshold, sensitivity, specificity, and AUC, was used to assess the predictive ability of individual indicators in the development of expected outcomes. Youden’s test, including both sensitivity and specificity, was used to determine the best parameter for the task.

## 5. Conclusions

The impaired L-arginine-eNOS-NO functional system was associated with impaired erythrocyte disaggregation, increased BBB permeability, and development of WMH and CI in CSVD. Exceeding the erythrocyte disaggregation rate thresholds after incubation in L-arginine can be used to assess the functional state of the L-arginine-eNOS-NO system and identify persons with impaired NO bioavailability. L-arginine can be part of a CSVD therapeutic strategy to restrain CI.

## Figures and Tables

**Table 1:** Rheological properties of erythrocytes before (baseline) and after incubation with L-arginine (resultant) in CSVD patients and controls.

Rheological Characteristics	CSVD (n = 73)	Control (n = 19)	*p*-Value
**Amp** (relative units)			
baseline	11.2 [9.5; 13.6]	12.0 [8.8; 13.9]	0.496
resultant	12.4 [10.6; 13.9]	12.5 [10.0; 14.6]	0.636
**Ts** (s)			
baseline	14.1 [12.0; 16.9]	19.1 [14.0; 25.6]	**0.005**
resultant	15.6 [12.4; 22.4]	19.8 [14.4; 25.9]	**0.028**
**Tf** (s)			
baseline	2.0 [1.5; 2.3]	2.7 [1.7; 3.9]	**0.023**
resultant	2.2 [1.6; 3.0]	3.2 [2.2; 3.8]	**0.006**
**AI** (%)			
baseline	65.2 [61.2; 69.3]	58.4 [52.0; 69.1]	**0.030**
resultant	61.0 [55.3; 67.4]	55.3 [50.3; 61.3]	**0.011**
**y**-**dis** (s^−1^)			
baseline	195 [140; 250]	125 [100; 180]	**0.003**
resultant	160 [100; 200]	100 [100; 110]	**0.002**
**DImax**			
baseline	0.42 [0.38; 0.48]	0.43 [0.36; 0.48]	0.993
resultant	0.34 [0.28; 0.38]	0.35 [0.28; 0.38]	0.817

Notes: CSVD, cerebral small vessel disease; Amp, aggregation amplitude; Ts, aggregation first phase time; Tf, aggregation second phase time; AI, aggregation index; y-dis, disaggregation rate; DImax, erythrocyte deformability.

**Table 2:** Resultant (after incubation with L-arginine) rheological characteristics of erythrocytes.

Parameter	Area under the Curve (CI)	Threshold	Sensitivity	Specificity	Odds Ratio (CI)
Amp (relative units)	0.535 (0.388–0.682)	12.45	53%	51%	1.07 (0.88–1.30)
Ts (s)	0.664 (0.537–0.823)	187	63%	66%	0.99 (0.88–1.10)
Tf (s)	0.703 (0.584–0.823)	2.7	63%	63%	1.65 (0.47–5.79)
AI (%)	0.690 (0.562–0.818)	59.3	60%	74%	1.03 (0.84–1.26)
y-dis (s^−1^)	0.733 (0.609–0.856)	112.5	67%	79%	0.98 (0.97–1.00)
DImax	0.517 (0.375–0.660)	0.345	53%	52%	0.15 (0.00–97.28)

Notes: Amp, aggregation amplitude; Ts, aggregation first phase time; Tf, aggregation second phase time; AI, aggregation index; y-dis, disaggregation rate; DImax, RBC deformability.

**Table 3:** Clinical characteristics of groups with y-dis < 112 s^−1^ and >113 s^−1^.

Parameters	y-dis ≤ 112 s^−1^	y-dis ≥ 113 s^−1^	*p*-Value
	(n = 39)	(n = 53)	
Gender, women (n, %)	26 (66.6%)	36 (67.9%)	0.951
Age, years (mean ± SD)	59.1 ± 6.0	59.7 ± 7.0	0.626
AH (n, %)	25 (64.1%)	45 (84.9%)	**0.001**
Degree of AH (n, %)			**0.011**
Grade 1	11 (44.0%)	8 (17.8%)	
Grade 2	7 (27.0%)	8 (17.8%)	
Grade 3	7 (27.0%)	29 (64.4%)	
DM type 2 (n, %)	1 (2.3%)	14 (26.4%)	**0.002**
Hypercholesterolemia	21 (58.5%)	27 (50.9%)	0.154
(total cholesterol > 6.2 mmol/L or statin use) (n, %)			
Obesity (body mass index > 30 kg/m²) (n, %)	11 (28.2%)	28 (52.8%)	**0.021**
Smoking (n, %)	13 (33.3%)	14 (26.4%)	0.643
WMH, Fazekas score (n, %)			
Score 0	15 (38.5%)	4 (7.6%)	**0.001**
Score 1	9 (23.1%)	9 (16.9%)	
Score 2	7 (17.9%)	18 (33.9%)	
Score 3	8 (20.5%)	22 (41.6%)	
MoCA scale (median [IQR])	28 [25; 29]	25 [22; 26]	**<0.001**

Notes: CSVD, cerebral small vessel disease; AH, arterial hypertension; DM, diabetes mellitus; WMH, white matter hyperintensity; MoCA, Montreal Cognitive Assessment; y-dis, disaggregation rate.

**Table 4:** BBB permeability by DCE-MRI in groups with y-dis < 112 s^−1^ and >113 s^−1^.

Parameter	y-dis < 112 s^−1^ (n = 39)	y-dis > 113 s^−1^ (n = 53)	*p*-Value
Ktrans GM	0.0002 [0.0001; 0.0004]	0.0002 [0.0001; 0.0003]	0.695
Vp GM	1.1267 [0.8214; 1.3489]	1.2837 [1.0199; 1.5810]	0.090
AUC GM	0.0026 [0.0020; 0.0031]	0.0033 [0.0026; 0.0037]	**0.019**
Ktrans NAWM	0.0001 [0.0000; 0.0001]	0.0000 [0.0000; 0.0001]	0.421
Vp NAWM	0.4395 [0.3835; 0.5937]	0.5531 [0.4167; 0.6281]	0.121
AUC NAWM	0.0011 [0.0009; 0.0012]	0.0013 [0.0011; 0.0015]	**0.007**
Ktrans WMH	0.0001 [0.0000; 0.0002]	0.0001 [0.0000; 0.0002]	0.688
Vp WMH	0.4541 [0.3118; 0.6081]	0.4758 [0.3320; 0.7470]	0.695
AUC WMH	0.0010 [0.0008; 0.0016]	0.0012 [0.0010; 0.0015]	0.334

Notes: Ktrans, BBB permeability coefficient; Vp, partial plasma volume; AUC, area under the curve corresponding to the increase in the time of contrast passage through the BBB; WMH, white matter hyperintensity; NAWM, normal-appearing white matter; GM, grey matter; y-dis, disaggregation rate.

**Table 5:** Comparison of indicators of vascular damage, nitric oxide, and its metabolites in groups with y-dis < 112 s^−1^ and >113 s^−1^.

Parameter	y-dis < 112 s^−1^ (n = 39)	y-dis > 113 s^−1^ (n = 53)	*p*-Value
NO3 (μmol/L)	58 [45; 76]	52 [32; 74]	0.139
NO2 (μmol/L)	49 [34; 68]	43 [24; 67]	0.127
NO (μmol/L)	9 [7; 13]	9 [5; 12]	0.240
TNF-α, pg/mL	25.0 [18.3; 44.0]	26.0 [18.5; 41.0]	0.937
TGF-β1, ng/mL	2.70 [2.25; 4.80]	3.15 [2.46; 5.85]	0.200
VEGF-A, pg/mL	212 [160; 411]	248 [109; 400]	0.928
HIF-1α, ng/mL	0.5 [0.36; 0.65]	0.48 [0.38; 0.68]	0.861
FGF, pg/mL	300 [250; 420]	340 [255; 438]	0.730
PAI-1, ng/mL	25.5 [16.4; 34.2]	25.2 [17.1; 35.9]	0.983
t-PA, ng/mL	3.20 [0.93; 8.80]	6.5 [2.75; 10.40]	0.021

Notes: NO, nitric oxide; TNF-α, tumor necrosis factor alpha; TGF-β1, transforming growth factor beta-1; VEGF-A, vascular endothelial growth factor-A; HIF-1α, hypoxia-inducible factor 1-alpha; FGFs, fibroblast growth factors; PAI-1, plasminogen activator inhibitor; t-PA, tissue plasminogen activator; y-dis, disaggregation rate.

**Table 6:** Description of erythrocytes’ rheological characteristics.

Designation	Parameter	Description
Amp (relative units)	Syllectogram amplitude	A parameter showing the overall degree of aggregation
Ts (s)	The formation time of coin erythrocyte columns	Erythrocyte 3D aggregate formation contribution, result of curve fit
Tf (s)	The formation time of erythrocyte three-dimensional aggregates	Erythrocyte rouleaux formation time constant, result of curve fit
AI (%)	Aggregation index	A calculated parameter that includes the phases of aggregation and disaggregation, thus reflecting the entire process
y-dis (s^−1^)	Disaggregation complete rate	A parameter that reflects the force required to destroy erythrocyte aggregates, thus reflecting the density of erythrocyte aggregates
DImax *	Erythrocyte deformability	Evaluation of the stability of erythrocyte membranes based on the calculation of the elongation index and the correlation of the time of exposure to shear stress

Notes: DImax *—elongation index. A similar name, EImax, may be used in the literature (the name depends on the version of LORRCA).

## Data Availability

Raw data were generated at the Research Center of Neurology. The data that support the findings of this study are available from the corresponding author upon reasonable request. Clinical, neuroimaging, laboratory, and statistical data will be available upon request from any qualified investigator.

## Appendix A

**Table A1:** Clinical characteristics of CSVD patients and controls.

Parameters	CSVD (n = 73)	Control (n = 19)	*p*-Value
Gender, women (n, %)	48 (65.8%)	14 (73.7%)	0.592
Age, years (mean ± SD)	60.1 ± 6.5	57.9 ± 6.4	0.158
AH (n, %)	65 (89.0%)	9 (47.4%)	<0.001
Degree of AH (n, %) Grade 1 Grade 2 Grade 3	10 (15.4%) 16 (24.6%) 39 (60.0%)	5 (55.6%) 3 (33.3%) 1 (11.1%)	<0.001
DM type 2 (n, %)	15 (20.5%)	0 (0%)	0.034
Hypercholesterolemia (total cholesterol > 6.2 mmol/L or statin use) (n, %)	39 (53.4%)	9 (47.4%)	0.188
Obesity (body mass index > 30 kg/m²) (n, %)	34 (46.6%)	5 (26.3%)	0.127
Smoking (n, %)	19 (26.0%)	8 (42.1%)	0.147
WMH, Fazekas score (n, %) Score 0 Score 1 Score 2 Score 3	73 (100%) 18 (24.7%) 25 (34.2%) 30 (41.1%)	0 (0%) 0 (0%) 0 (0%) 0 (0%)	<0.001
MoCA scale (median [IQR])	25 [22; 26]	29 [27; 30]	<0.001

Notes: CSVD, cerebral small vessel disease; AH, arterial hypertension; DM, diabetes mellitus; WMH, white matter hyperintensity; MoCA, Montreal Cognitive Assessment.

**Table A2:** BBB permeability by DCE-MRI in CSVD patients and controls.

Parameter	CSVD (n = 73)	Control (n = 19)	*p*-Value
Ktrans GM	0.0002 [0.0001; 0.0004]	0.0002 [0.0001; 0.0002]	0.204
Vp GM	1.2750 [1.0199; 1.5869]	0.9809 [0.8138; 1.3205]	0.035
AUC GM	0.0031 [0.0025; 0.0037]	0.0022 [0.0019; 0.0026]	0.001
Ktrans NAWM	0.0001 [0.0000; 0.0001]	0.0000 [0.0000; 0.0001]	0.628
Vp NAWM	0.5293 [0.4076; 0.6449]	0.4248 [0.3690; 0.4831]	0.047
AUC NAWM	0.0013 [0.0011; 0.0015]	0.0010 [0.0009; 0.0012]	0.002
Ktrans WMH	0.0001 [0.0000; 0.0002]	─	─
Vp WMH	0.4734 [0.3320; 0.6180]	─	─
AUC WMH	0.0012 [0.0008; 0.0015]	─	─

Notes: Ktrans, BBB permeability coefficient; Vp, partial plasma volume; AUC, area under the curve corresponding to the increase in the time of contrast passage through the BBB; WMH, white matter hyperintensity; NAWM, normal-appearing white matter; GM, grey matter.

**Table A3:** Comparison of indicators of vascular damage, nitric oxide, and its metabolites in CSVD patients and controls.

Parameter	CSVD (n = 73)	Control (n = 19)	*p*-Value
NO3 (μmol/L)	52 [32; 66]	73 [55; 85]	0.004
NO2 (μmol/L)	43 [25; 55]	58 [48; 72]	0.005
NO (μmol/L)	8 [6; 12]	11 [6; 16]	0.364
TNF-α, pg/mL	30.0 [19.0; 49.0]	21.5 [14.0; 28.0]	0.028
TGF-β1, ng/mL	3.00 [2.40; 5.70]	2.60 [1.74; 3.45]	0.027
VEGF-A, pg/mL	212 [98; 352]	320 [182; 420]	0.049
HIF-1α, ng/mL	0.43 [0.34; 0.65]	0.55 [0.50; 0.65]	0.029
FGF, pg/mL	310 [240; 460]	328 [265; 410]	0.705
PAI-1, ng/mL	25.1 [16.8; 35.8]	25.6 [12.5; 31.0]	0.598
t-PA, ng/mL	5.90 [1.30; 9.70]	3.80 [0.93; 7,50]	0.137

Notes: NO, nitric oxide; TNF-α, tumor necrosis factor alpha; TGF-β1, transforming growth factor beta-1; VEGF-A, vascular endothelial growth factor-A; HIF-1α, hypoxia-inducible factor 1-alpha; FGFs, fibroblast growth factors; PAI-1, plasminogen activator inhibitor; t-PA, tissue plasminogen activator.
